# Atraumatic Splenic Rupture Associated with Influenza A (H1N1) Pneumonia: Case Report and Review of the Literature

**DOI:** 10.1155/2021/6516064

**Published:** 2021-08-03

**Authors:** Dilraj Deol, Huimin Wu, Anayansi Lasso-Pirot, Kathryn S Robinett, Montserrat Diaz-Abad

**Affiliations:** ^1^Department of Medicine, MedStar Good Samaritan Hospital, Baltimore, Maryland, USA; ^2^Department of Medicine, University of Maryland Saint Joseph Medical Center, Towson, Maryland, USA; ^3^Department of Pediatrics, University of Maryland School of Medicine, Baltimore, Maryland, USA; ^4^Department of Medicine, University of Maryland School of Medicine, Baltimore, Maryland, USA

## Abstract

Influenza virus infection may present with fever, chills, headache, myalgia, malaise, and respiratory symptoms, with a few cases developing into pneumonia, respiratory failure, and other organ damage. Very few cases of atraumatic splenic rupture associated with influenza infection have been reported. Atraumatic splenic rupture, while rare, is associated with high mortality. Here, we report the first case of atraumatic splenic rupture associated with influenza infection in the English literature and review the prior reported literature. The patient was diagnosed with influenza A (H1N1) pneumonia and subsequently developed hemorrhagic shock requiring emergency laparotomy and removal of the ruptured spleen.

## 1. Introduction

Influenza viruses are divided into three types, influenza A, B, and C viruses. Influenza A viruses cause epidemic influenza and sporadic pandemics, and they are further classified into subtypes on the basis of the antigenic properties of two surface glycopeptides, hemagglutinin (H) and neuraminidase (N). There are 18 different H subtypes and 11 different N subtypes (H1 to H18 and N1 to N11). Influenza A (H1N1), which caused a world pandemic in 2009, is one of the common influenza viruses and is now included in each year's influenza vaccine [1].

Influenza infection may present with fever, chills, headache, myalgia, malaise, and anorexia, accompanied by respiratory symptoms, including nonproductive cough, nasal discharge, and sore throat [1]. Some patients may develop pneumonia, respiratory failure, and other organ damage [2].

Very few cases [3–7] of atraumatic splenic rupture associated with influenza infection have been reported in the literature. Atraumatic splenic rupture, is rare and, if missed, can be associated with high mortality [8]. We report the first case in the English literature of atraumatic splenic rupture associated with influenza infection and review the prior reported literature.

## 2. Case Presentation

A 50-year-old man with past medical history of hypothyroidism and cigarette smoking had been on vacation in Florida, USA, with his family for 3 weeks. The day of his return flight in July, he developed a sore throat and body aches followed by fever and a productive cough. He went to urgent care, was diagnosed with acute bronchitis, and was treated as outpatient with azithromycin and prednisone for 5 days without improvement. On the eighth day of symptoms, he developed confusion and was brought to the emergency department (ED) for further evaluation.

In the ED, the patient had mild shortness of breath but denied nausea, vomiting, or abdominal pain. On physical exam, he had temperature 38.3°C, heart rate 120 beats/min, respiratory rate 25 breaths/min, and SpO_2_ 90% on room air, with coarse breath sounds bilaterally on lung exam; he was started on oxygen 2 L/min via nasal cannula. A chest X-ray showed bibasilar lung infiltrates. Testing revealed white blood cell count 9.0 k/uL (4.0–10.0 k/uL), hemoglobin (Hb) 15.8 g/dL (13.0–18.0 g/dL), platelets 249 k/uL (150–400 k/uL), international normalized ratio 1.4 (≤3.5), protime 15.1 seconds (10.0–12.0 seconds), partial thromboplastin time 37.4 seconds (25.0–35.0 seconds), blood urea nitrogen 28 mg/dL (9–20 mg/dL), creatinine 1.46 mg/dL (0.66–1.25 mg/dL), total protein 7.5 g/dL (6.3–8.2 g/dL), albumin 3.9 g/dL (3.5–5.0 g/dL), total bilirubin 1.4 mg/dL (0.2–1.3 mg/dL), alkaline phosphatase 195 U/L (38–126 U/L), alanine aminotransferase 160 U/L (21–72 U/L), and aspartate aminotransferase 243 U/L (17–59 U/L). Urine *Legionella* antigen and *Streptococcus pneumoniae* antigen tests were negative. An ultrasound of the abdomen showed a contracted gallbladder with probable calculi and normal pancreas and spleen. He was diagnosed with community acquired pneumonia, started on intravenous ceftriaxone and azithromycin, and admitted to the medical floor. He was also started on heparin 5000 U subcutaneously every 8 hours for thromboembolism prophylaxis.

The patient's shortness of breath deteriorated overnight. Chest X-ray showed worsening lung infiltrates, predominantly in the left lung. He had increased work of breathing and worsening hypoxemia requiring oxygen via high flow nasal cannula and transfer to the intensive care unit. More information was obtained from the family: his wife and father also had developed a cough and no one in the family including the patient had ever received influenza vaccination. A polymerase chain reaction (PCR) respiratory viral panel on a nasopharyngeal swab sample was positive for influenza A (H1N1) virus, and therapy was started with oseltamivir.

**Figure 1:**
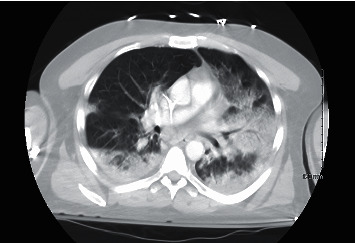
Computed tomography scan (CT) of the chest showing infiltrates in both lower lung lobes.

**Figure 2:**
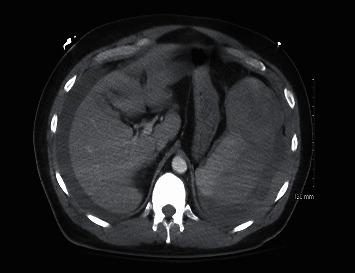
Initial computed tomography scan (CT) of the abdomen showing perihepatic and perisplenic fluid, and the lateral margin of the spleen indistinct with low density areas, concerning for splenic injury or rupture.

The patient's respiratory status continued to worsen. He developed hypotension and severe abdominal pain on the third day of hospitalization. Serum amylase was 157 U/L (30–110 U/L) and lipase 1454 U/L (23–300 U/L). Hb decreased from 15.8 to 7.4 g/dL. Coagulation parameters were normal. Arterial blood gases revealed pH 7.5 (7.35–7.45), PaCO_2_ 21 mm Hg (35–45 mm Hg), and PaO_2_ 52 mm Hg (80–100 mm Hg) on high flow nasal cannula FiO_2_ 70% at 50 L/min and the patient was subsequently intubated. Computed tomography (CT) of the chest and abdomen revealed lung consolidation of the left upper lobe and infiltrates in both lower lobes and right middle lobe, with no evidence of pulmonary embolism (Figure 1). The lateral margin of the spleen was indistinct and with low density areas, concerning for splenic injury or rupture (Figure 2). The patient denied any prior abdominal trauma.

**Figure 3:**
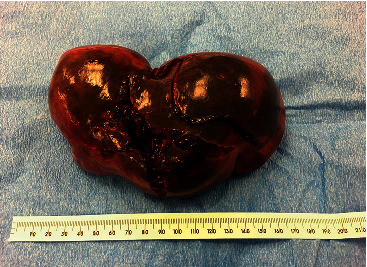
Appearance of the spleen after splenectomy. The capsular surface was hemorrhagic with an irregular 10 cm laceration in the mid portion nearly bisecting the spleen with an intact hilum.

Due to these findings along with suspected ongoing hemorrhage with decreasing Hb values since admission, the patient underwent an embolization of the splenic artery by interventional radiology. Despite this procedure, Hb values continued to decrease, remaining at 7.7 g/dL despite transfusion with 3 units of packed red blood cells. The patient underwent emergency laparotomy with splenectomy to control the hemorrhage. During the surgery, there was extensive hemoperitoneum and the spleen capsular surface was found to be hemorrhagic with a large laceration in the mid portion (Figure 3). Pathologic examination revealed a spleen with weight 521 g and size 15 cm × 10 cm × 6 cm. The capsular surface was deep red due to hemorrhage with an irregular 10 cm laceration in the mid portion nearly bisecting the specimen with an intact hilar margin. There was a slightly puckered area located 5.5 cm from the point of laceration with an underlying somewhat stellate area of apparent fibrous tissue with vascular proliferation consistent with a hemangioma measuring 1.5 cm in greatest dimension. The splenic parenchyma was red brown. There was a large hematoma with a focal rim of tan white fibrous tissue. There was no evidence of malignancy.

A bronchoscopy with bronchoalveolar lavage was performed in the operating room after the splenectomy, and the culture was positive for influenza A (H1N1) virus only. Infectious mononucleosis and malaria work up were negative. The patient was continued on oseltamivir for treatment of influenza pneumonia and acute hypoxemic respiratory failure, was extubated successfully after 14 days of mechanical ventilatory support, and was discharged home in good condition after 40 days of hospitalization.

## 3. Discussion

We present the case of a patient with severe influenza A (H1N1) pneumonia and respiratory failure, who experienced atraumatic splenic rupture requiring emergency splenectomy to control hemorrhagic shock. While associated with other viral infections, atraumatic splenic rupture has been very rarely reported in association with influenza infection, and to our knowledge, never in the English literature as the only associated condition.

In addition to its common symptoms, some patients with influenza A (H1N1) virus infection develop severe disease, complicated with pneumonia, acute respiratory distress syndrome, respiratory failure, and other organ damage such as myositis, rhabdomyolysis, renal failure, myocarditis, pericarditis, encephalopathy, and liver function abnormalities [2]. In addition to pneumonia and respiratory failure, our patient experienced acute renal failure and transaminitis, as well as an atraumatic splenic rupture with hemorrhagic shock, requiring multiple transfusions and emergency splenectomy after a failed splenic artery embolization. Splenic rupture can be life-threatening and requires a high index of clinical suspicion and emergent intervention to prevent death. Since the spleen plays a key role in combating infection, a conservative, interventional, or spleen-preserving surgical approach should be attempted first. However, total splenectomy should be performed for patients with severe rupture, hemodynamic instability, or continued and recurrent bleeding [9].

Atraumatic splenic rupture is rare. Common causes include infection, neoplasms, and vasculitis [8, 10]. One large systematic review reported 613 cases of splenic rupture without risk factors or previously diagnosed diseases [8]. Malaria, mononucleosis, cytomegalovirus infection, and typhoid fever were common infectious causes. Only 3 cases were associated with pneumonia, 2 of them caused by *Legionella* infection. Influenza infection was not reported in that study. Another systematic review reported 845 patients with atraumatic splenic rupture [10]; of those, 137 patients had an infectious cause, but none had influenza infection.

The precise pathologic mechanisms implicated in atraumatic splenic rupture have not been conclusively determined and probably vary from case to case. Mechanisms that have been proposed in different cases include (1) increased intra-abdominal pressure (related to coughing, sneezing, emesis, defecation, and pregnancy) leading to accumulated trauma on the splenic capsule; (2) an increase in intrasplenic tension due to cellular hyperplasia and vascular engorgement; and (3) reticular endothelial hyperplasia resulting in thrombosis and infarction, leading to hemorrhage and rupture of the splenic capsule. In addition, coagulopathy, thrombocytopenia, and platelet dysfunction may prolong hemorrhage that would otherwise be self-limited [11, 12].

It is possible that an exaggerated immune response to influenza infection may play a role in this rare complication. The influenza A (H1N1) virus has been isolated on organ samples of the lung, brain, kidney, and spleen on animal experiments, [13] and it has also been reported that the spleen plays a particularly active role in the innate immune response to influenza A (H1N1) [14]. The precise mechanisms involved in this case are unclear, but there was a productive cough present which could have contributed to the rupture and the patient was also receiving a prophylactic dose of heparin. While a very small hemangioma was noted on histologic examination, there was no evidence of bleeding in its location.

So far, there have been at least 5 other cases reported of atraumatic splenic rupture associated with influenza infection, none in the English literature. The first case was in Russia (former Soviet Union) in 1963 [3]. A 43 y/o woman with severe influenza reported mild pain in the left upper abdomen. She was treated as an outpatient and recovered completely. One month after the symptoms started, she developed severe abdominal pain in the same area and fever 38°C. Four days later, she was admitted to the hospital with severe diffuse abdominal pain, nausea, vomiting, lightheadedness, fever 38.5°C, and unmeasurable blood pressure. On exam, she was very pale, the abdomen was distended and tender, and a fluid shift was present. She was treated for hemorrhagic shock and underwent emergency laparotomy with splenectomy. Surgical findings were an enlarged ruptured spleen and 1.5 L of blood and clots in the abdominal cavity. On pathology, the spleen was congested with blood, with lymphoid hyperplasia and necrotic tissue around the rupture. The patient recovered.

The second case occurred in Poland in 1964 [4]. A 22-year-old male had influenza and related symptoms for 10 days. One day after admission, he developed severe pain in the left epigastric and lumbar regions and anemia that did not respond to transfusion. On the sixth day, the pain increased, and the patient underwent a laparotomy with splenectomy. An enlarged, necrosed spleen was noted with blood clots surrounding it and 1 L of blood in the peritoneal cavity. He recovered.

A third case was reported in Ukraine (former Soviet Union) in 1969 [5]. A 60-year-old woman was admitted with influenza pneumonia confirmed by antibody titers. She had severe headache, fever 38.1°C, cough, and rhinorrhea. Over the next four days, all symptoms resolved, but she then developed acute, severe pain in the epigastric area, nausea, and vomiting. Blood pressure was 80/40 mm Hg, with severe tachycardia and Hb 5.2 g/dL. The patient underwent emergency laparotomy for worsening shock. During the surgery, 2 L of blood and clots were found in the abdominal cavity along with a ruptured spleen requiring splenectomy. On pathology of the spleen, there was significant sclerosis of the red pulp and of the stroma surrounding the follicles. Almost all vessels were significantly narrowed with thickened walls; parts of the vessel walls had necrotic changes and there was cellular debris in some follicles. The patient was discharged home 2 months later. Of note, the article references one additional case of splenic rupture with influenza authored by PM Geyler in 1963; full reference was not provided, and the report was not able to be located despite a comprehensive search.

A fourth case was published in Hungary in 1979 [6]. A 57-year-old man with influenza had experienced 7 days of fever and limb pain at home and was already improving. He then developed weakness and dizziness and was admitted to the hospital. He had abdominal pain, Hb 9.9 g/dL, tachycardia, and hypotension. He underwent laparotomy where a large amount of clotted blood was found in the abdominal cavity. The spleen had a transverse rupture of 1.5 cm on its anterior surface, which did not penetrate the entire thickness and a splenectomy was done. On gross examination, the spleen weighed 160 g and demonstrated a 3 cm disruption laterally in the lower third extending 5–6 mm into the parenchyma. On microscopy, the follicular pattern was preserved with prominent germinal centers. Areas of parenchymal hemorrhage were also noted. The histological picture was thought to be similar to changes seen in sepsis, which suggested to the authors the possibility of a viral infection as the cause.

A fifth case was published in Spain in 2011 [7]. A 36-year-old man had fever 39.5°C, vomiting, diarrhea, and epigastric pain for 5 days and was admitted. On exam, he had blood pressure 95/55 mm Hg and heart rate 100 beats/min. One day later, the patient had intense abdominal pain and lost consciousness when standing. His Hb was 8.9 g/dL and an abdominal CT showed lung alveolar infiltrate in the bases and a splenic rupture with active hemorrhage and hemoperitoneum. A laparotomy with splenectomy was done. On pathology, the spleen was of normal size with the external surface showing some hemorrhagic areas with adherent clots that covered less than 5% of the external surface. A histologic diagnosis of capsular rupture with hemorrhagic foci over a normal spleen was made. After the urgent surgery, the patient recovered and was later diagnosed with influenza A (H1N1) pneumonia with a positive PCR test.

The above mentioned 5 cases, except for the last one, were reported more than 40 years ago. Reports were brief, with limited information provided including figures and diagnostic methods and with outdated terminology. They do, however, have all in common a diagnosis of splenic rupture without a history of prior trauma and an associated diagnosis of influenza, with the influenza-related symptoms preceding the abdominal signs and symptoms, anemia, and hemorrhagic shock associated with the splenic rupture.

Besides the abovementioned cases, there is one reported case in the English literature of atraumatic splenic rupture associated with influenza A (H1N1) infection. However, this case also had coinfection with *Legionella pneumonia* [15], which has also been associated with splenic rupture [8], so it cannot be ruled out that the splenic rupture could be due to *Legionella* infection instead of the influenza infection, or due to coinfection. The patient was a 42-year-old man who was hospitalized with fever of 39°C, progressive dyspnea, and nonproductive cough. On admission, he had anemia with Hb of 5 g/dL and tested positive both for influenza A (H1N1) virus by PCR test on a throat swab and for *Legionella* by urinary antigen test and PCR in peripheral blood. Total body CT showed extensive bilateral interstitial pneumonia and signs of incipient splenic rupture. He underwent laparotomy with splenectomy. Histological examination showed diffuse splenic intravascular coagulation. The patient achieved full clinical recovery.

In conclusion, we present a very rare case of atraumatic splenic rupture associated with influenza A (H1N1) infection, which required emergency laparotomy with splenectomy to control hemorrhage after a failed splenic artery embolization. To our knowledge, this is the first case reported in the English literature of this complication associated with influenza infection, and only one of a handful reported previously worldwide. Clinicians should be aware of this very rare but potentially fatal complication of influenza infection, in particular in patients who develop abdominal signs and symptoms, unexplained anemia, and hemodynamic instability.

## Data Availability

No data were used to support this study.
